# Neo-adjuvant chemo-radiation of rectal cancer with Volumetric Modulated Arc Therapy: summary of technical and dosimetric features and early clinical experience

**DOI:** 10.1186/1748-717X-5-14

**Published:** 2010-02-19

**Authors:** Antonella Richetti, Antonella Fogliata, Alessandro Clivio, Giorgia Nicolini, Gianfranco Pesce, Emanuela Salati, Eugenio Vanetti, Luca Cozzi

**Affiliations:** 1 Oncology Institute of Southern Switzerland, Radiation-Oncology Dept, Bellinzona, Switzerland; 2 Oncology Institute of Southern Switzerland, Medical Physics Unit, Bellinzona, Switzerland

## Abstract

**Background:**

To report about initial technical and clinical experience in preoperative radiation treatment of rectal cancer with volumetric modulated arcs with the RapidArc^® ^(RA) technology.

**Methods:**

Twenty-five consecutive patients (pts) were treated with RA. All showed locally advanced rectal adenocarcinoma with stage T2-T4, N0-1. Dose prescription was 44 Gy in 22 fractions (or 45 Gy in 25 fractions). Delivery was performed with single arc with a 6 MV photon beam. Twenty patients were treated preoperatively, five did not receive surgery. Twenty-three patients received concomitant chemotherapy with oral capecitabine. A comparison with a cohort of twenty patients with similar characteristics treated with conformal therapy (3DC) is presented as well.

**Results:**

From a dosimetric point of view, RA improved conformality of doses (CI_95% _= 1.1 vs. 1.4 for RA and 3DC), presented similar target coverage with lower maximum doses, significant sparing of femurs and significant reduction of integral and mean dose to healthy tissue. From the clinical point of view, surgical reports resulted in a down-staging in 41% of cases. Acute toxicity was limited to Grade 1-2 diarrhoea in 40% and Grade 3 in 8% of RA pts, 45% and 5% of 3DC pts, compatible with known effects of concomitant chemotherapy. RA treatments were performed with an average of 2.0 vs. 3.4 min of 3DC.

**Conclusion:**

RA proved to be a safe, qualitatively advantageous treatment modality for rectal cancer, showing some improved results in dosimetric aspects.

## Background

Pre-operative chemo-radiotherapy of rectal cancer in locally advanced stage has become a widely accepted treatment modality as reported by Roh et al [1] or by Gollins et al [2] and references there-in. Locally advanced rectal cancer treated with neoajuvant chemoradiation therapy is expected to show positive response with tumour downstaging in about 45%-47% of patients [3,4]. Although no effective method has been identified so far to predict outcome from molecular biomarkers or other methodology, research is actively performed to identified clinically valuable predictors (e.g. serum carinoembryonic antigen was reported to be predictor of pathologic tumour response by Yoon et al. [4]). Hystopatological downstaging was also reported to be poorly correlated with tumor volume reduction as measured with magnetic resonance imaging after treatment [5]. The management of advanced rectal cancer is generally approached with two different radiotherapy scheduling, with a short or a long course for preoperative treatments [6]. Longer courses have been more frequently adopted for advanced stages and, although presenting, on the tolerance side, a higher rate of reversible acute toxicity, these schemes showed lower rate of late gastrointestinal toxicity. A Phase I trial with hypofractionated intensity-modulated radiotherapy and simultaneous boost in association with capecitabine chemotherapy was reported as unacceptably toxic and was interrupted [7] questioning at the same time the role of advanced treatment modalities like intensity modulation and the application of alterated fractionations to locally advanced rectal cancer.

In summary, the current challenge is to improve outcomes whereas minimizing morbidity and maximizing the potential for more complete surgery resection and sphincter preservation.

Aim of the present study is to report the technical and dosimetric aspects of the treatments as well as to summarize early acute toxicity findings and histo-pathological results after surgery. The introduction of an advanced technology as volumetric modulated arc therapy in clinical practice follows the hypothesis that this shall lead to dosimetric and clinical results not inferior and possibly superior to previous treatment modalities. For this reason, to prove the hypothesis at a basic level, as a first approach to the new treatment modality, the first group of patients treated with this new modality at our institute, was planned with the same planning objectives of the previously adopted conformal modality, in order to demonstrate at least the clinical equivalence of the new approach with conformal data, and to possibly give information about the potential improvements on toxicity rates or on logistic side. To enable a qualitative comparison of the treatment features and early outcome, a group of patients with similar characteristics but irradiated with a 3D conformal technique, was pooled out of the institutional database and analysed in parallel. A similar investigaton was performed at planning level by Engels et al [8] comparing Helical Tomotherapy and 3D-conformal plans showing that the combination of helical tomotherapy and in their case the usage of daily adaped margins based on MV-CT imaging significantly decreases the probability of gastro-intestinal toxicity. Consistently, a study from the William Beaumont Hospital group [9] showed a clear and highly significant dose-volume relationship between bowel irradiation and acute grade 3 diarrhoea suggesting the need of reducing as much as possible the involvement of the relatively distant organ at risk during pre-operative irradiation of rectal cancer. Similar results were reported by Tho et al. [10] from University of Glasgow showing a strong dose-volume relationship with acute diarrhoea at all dose levels. These results show that, although 3D conformal treatments are technically adequate for pre-operative rectal cancer patients, the application of advanced techniques shall be carefully explored being potentially beneficial for the patients.

The Volumetric Modulated Arc Therapy technique adopted for this study is RapidArc^® ^(RA), the technical solution realised by Varian Medical Systems (Palo Alto, California) and based on the original investigation of K. Otto [11].

RapidArc^® ^was recently introduced in clinical practice in several institutes after an intensive validation at planning level where it was compared to IMRT or other approaches, in a series of studies on brain tumours, prostate, head and neck, anal canal, cervix uteri and other indications [12-18]. At our institute, as per end of September 2009, 160 patients have been treated with RA for a variety of indications. Among these, twenty-five received RA treatment as part of their multidisciplinary management of rectal adenocarcinoma, most of them in pre-operative neo-adjuvant chemo-radiation setting. RA is implemented at planning level by means of the Progressive Resolution Optimisation (PRO) algorithm in the Eclipse planning system by Varian. The optimisation process is based on an iterative inverse planning process aiming to simultaneously optimise the instantaneous multi leaf collimator (MLC) positions, the dose rate, and the gantry rotation speed to achieve the desired dose distribution. Delivery is performed on Varian Clinacs as single or multiple arcs with dynamic continuous modulation of MLC shapes, gantry speed and dose rate.

### Materials and patients

Twenty-five consecutive patients presenting advanced rectal carcinoma were treated with RA from October 2008 to September 2009. A corresponding group of twenty patients, treated with 3D conformal therapy (3DC) during the years 2007 and 2008 was used as a benchmark; these patients were randomly pooled out of the institutional database. Comparison was performed against 3DC technique since this was the treatment modality in use for rectal patients before advent of volumetric modulated arc therapy at our institute. Random selection was performed from the entire group of rectal carcinoma patients restricting the selection to the same prescription, fractionation and combined chemotherapy regimen and to reproduce a reasonably similar group. The comparison with 3D conformal data was introduced to allow a qualitative appraisal of the consistency of RA based treatments with previous clinical experience in the same clinic without aiming to perform any equivalence study and without aiming to perform a matched pair analysis. Demographic and clinical characteristics of patients are summarized in table 1. Most of the patients were treated pre-operatively in a neo-adjuvant schedule with chemotherapy concomitant to radiation. Oral capecitabine was administered with standard dosage of 825 mg/mq twice a day. Four patients in the RA group were treated post-operatively and one did not receive surgery for patient's choice. Dominant stage was T3, N0-N1 and the main differentiation grade was G2. Median age was similar between the two groups (65.4 and 63.5 years respectively for RA and 3DC).

**Table 1:** Summary of patients characteristics at treatment start.

		RA	3DC
**Number of patients**		**25**	**20**

**Males**		76% (19/25)	65% (13/20)
**Females**		24% (6/25)	35% (7/20)

**Age [years] (median and range)**		65.4 [37, 85]	63.5 [46, 79]

**Hystology**		Adenoca	Adenoca

**Stage T**	**T2**	0% (0/25)	15% (3/20)
	**T3**	84% (21/25)	75% (15/20)
	**T4**	16% (4/25)	10% (2/20)

**Stage N**	**N0**	48% (12/25)	40% (8/20)
	**N1**	44% (11/25)	35% (7/20)
	**N2**	4% (1/25)	10% (2/20)
	**Nx**	4% (1/25)	15% (3/20)

**Stage M**	**M0**	96% (24/25)	95% (19/20)
	**M1**	4% (1/25)	5% (1/20)

**Grade G**	**G1**	4% (1/25)	10% 2/20)
	**G2**	84% (21/25)	90% (18/20)
	**G3**	12% (3/25)	0% (0/20)

**Location**	**Low**	48% (12/25)	15% (3/20)
	**Medium**	16% (4/25)	45% (9/20)
	**High**	36% (9/25)	40% (8/20)

**Chemotherapy**	**Capecitabin**	92% (23/25)	100% (20/20)
	**No chemio**	8% (2/25)	0% (0/20)

**Surgery**	**Pre-op**	80% (20/25)	90% (18/20)
	**Post-op**	16% (4/25)	10% (2/20)
	**Non-op**	4% (1/25)	0% (0/20)

**Radiation Dose Prescription**	**44 Gy/22 fractions**	72% (18/25)	80% (16/20)
	**45 Gy/25 fractions**	20% (5/25)	20% (4/20)
	**40 Gy/20 fractions**	8% (2/25)	0% (0/20)
**Radiation Boost**	**50.4 Gy**	12% (3/25)	25% (5/20)
**Dose Prescription**	**54.0 Gy**	4% (1/25)	0% (0/20)

Values are expressed in number of patients when not otherwise specified.

Gross Target Volume (GTV) was defined as the tumour and involved nodes visible to CT/MRI images. Clinical Target Volume (CTV) included the GTV with 1 cm isotropic margin and the pelvic lymph nodes that were not involved (peri-rectal, presacral, obturator, internal iliac). Planning target volume (PTV) was defined as the CTV with a margin of 8 mm. Organs at risk routinely considered in these patients were bladder, femoral heads and bowels (peritoneal region including small- and large- bowels excluding PTV). In addition, as defined for all patients treated with intensity modulated modalities, the Healthy Tissue (HT) was additionally defined as the patient's volume included in the CT dataset minus the PTV volume. This healthy tissue volume was generated also for the benchmark group of patients. Volumes are reported in the results section.

Dose prescription was set to either 44 Gy in 2 Gy/fractions or 45 Gy in 1.8 Gy/fractions for the majority of the cases (only 2 patients received 40 Gy in 2 Gy fractions in the RA group). Four (five) patients in the RA (3DC) group received also a final boost to 50.4 Gy (54 Gy in one case) being either post-operative or non operable cases. In all RA cases, dose normalization was set to mean dose to planning target volume (PTV) while for 3DC plans, dose normalization was set to isocentre, according to current and forthcoming ICRU recommendations.

RA plans were optimised for single arcs (rotation of 358°, from 179° to 181° CCW) for a Clinac 2100iX equipped with a Millennium-120 MLC (120 leaves with a resolution at isocentre of 5 mm for the inner 20 cm and 10 mm for the outer 2 × 10 cm) and a beam energy of 6 MV. Further details on RA technique can be found in [13,14]. Plan optimisation was performed requiring a PTV coverage of 95%-107%. Concerning OARs, bladder mean dose was required to be inferior to 35 Gy and V_40 Gy _< 50%. Maximum dose to femurs was not constrained.

3DC plans were designed, according to institutional practice, with three fields (one posterior and two laterals) with mechanical wedges on the lateral beams. Conformal shaping of the fields was performed by means of static MLC. Plans were computed for a Clinac 2100EX equipped with a MLC-80 (80 leaves with a resolution at isocentre of 10 mm) and for a beam energy of either 6 MV or 15 MV.

All dose distributions were computed with the Analytical Anisotropic Algorithm (AAA) implemented in the Eclipse planning system with a calculation grid resolution of 2.5 mm.

Technical features of treatments have been reported in terms of main delivery parameters (number of field or arcs, field or control point size, MU, MU/deg and MU/Gy, Dose Rate, Gantry speed, Collimator angle, beam-on and treatment time); beam-on and treatment times are defined without inclusion of patient positioning and imaging procedures and were automatically scored by the record and verify electronic system and derived offline from the database for this analysis. Beam on time includes only the time needed to deliver the required MU summed on all involved fields while treatment time includes machine set-up and programming time and time needed to move from one field to the next and to mount the mechanical wedges (for the 3DC technique). For RA, beam on time, with conventional fractionation, is bound to maximum angular speed of the gantry which is limited to 4.8°/sec; dose rate does not play a role in this case.

For RA patients, results of pre-treatment plan quality assurance are reported as Gamma Agreement Index (GAI), i.e. the percentage of modulated field area passing the γ-index criteria of Low [19] with thresholds on dose difference set to ΔD = 3% of the significant maximum dose, and on Distance to Agreement set to DTA = 3 mm. Measurements and analysis were performed by means of the GLAaS methodology described in [20,21] based on absorbed dose to water from EPID measurements. 3DC plans were not subject to pre-treatment dosimetric verification as normal practice for all non intensity modulated treatments, routine MU verification was performed on these patients.

Dosimetric quality of treatments was measured from dose volume histogram (DVH) analysis. For PTV the following data were reported: target coverage (D_1%_, D_99%_, V_95%_, V_107%_), homogeneity (D_5-95%_) and conformity (CI_90% _and CI_95%_). CI was defined as the ratio between the volume of patient irradiated at 95% (90%) of the prescribed dose and the PTV volume. For OARs, the mean dose, the maximum dose (D_1%_) and appropriate values of V_xGy _(volume receiving at least × Gy) were scored. For Healthy Tissue, the integral dose (DoseInt) was reported as well. This is measured as the integral of the dose delivered to the entire HT and is expressed in Gy cm^3^.

Wilcoxon non-parametric two-sample tests were applied to compute significance of observed dosimetric differences for the various parameters.

Clinical outcome of treatments was recorded in terms of observed acute toxicity, particularly incidence of disuria and diarrhoea. Toxicity scoring was assessed by non blind radiation oncologists in charge of the various patients and according to the National Cancer Institute Common Terminology Criteria of Adverse Effects scale (CTCAE version 3) as part of the routine visits during treatment and follow up protocols. At the time of surgery, histo-pathological reassessment of the tumour stage was performed. For the patients having this information available in the institutional database, the staging changement of the diseases was recorded, comparing data at surgery with data at diagnosis. Staging at diagnosis was assessed by means of standard diagnostic procedures including CT and MR imaging and clinical examination of patients.

## Results

**Figure 1:**
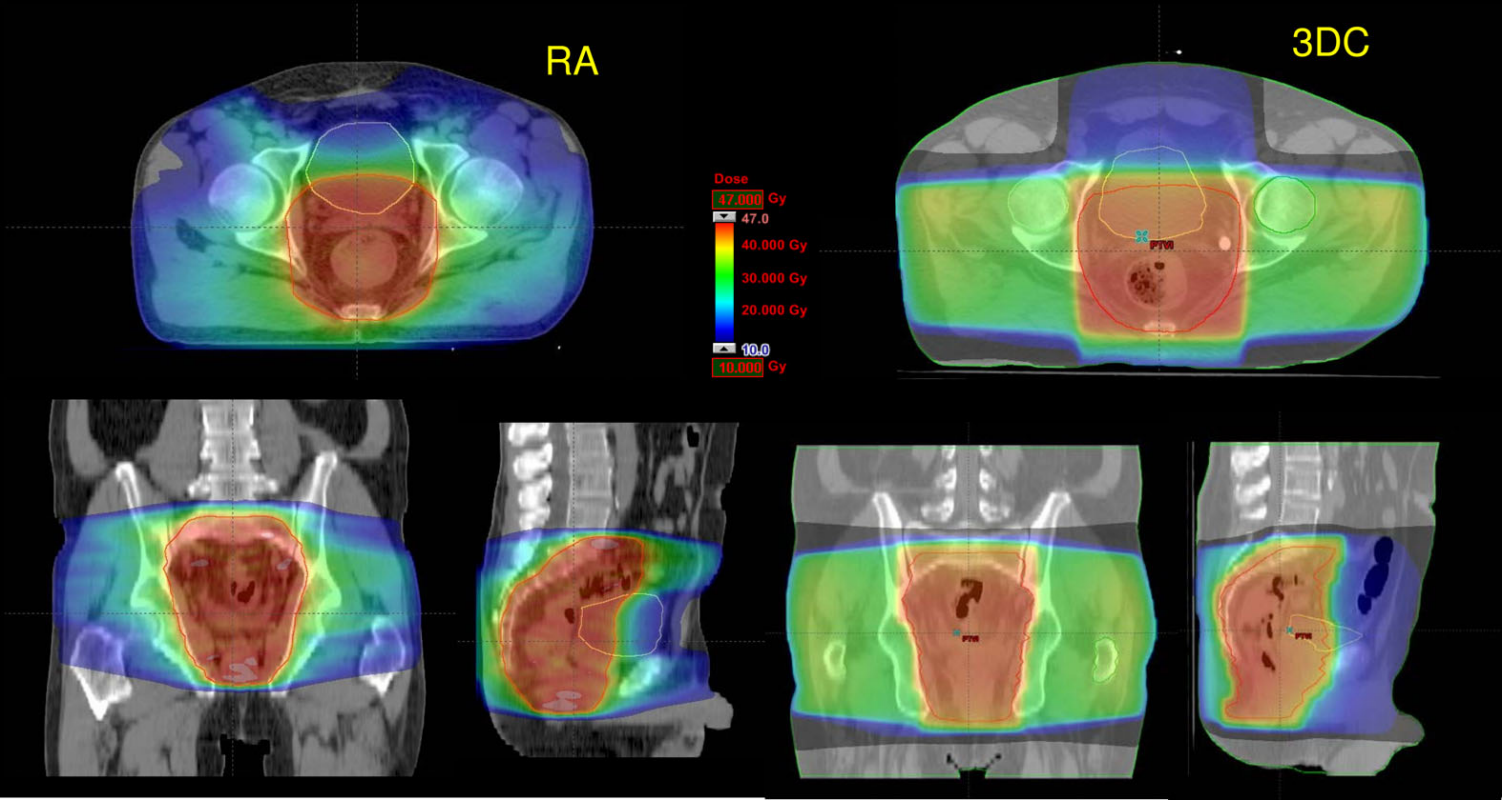
**Isodose distributions for two example patients for RA and 3DC treatments for an axial plane, sagittal and coronal views**. Doses are shown in colorwash within the interval from 10 to 47 Gy. PTV, bladder and femurs are outlined.

**Figure 2:**
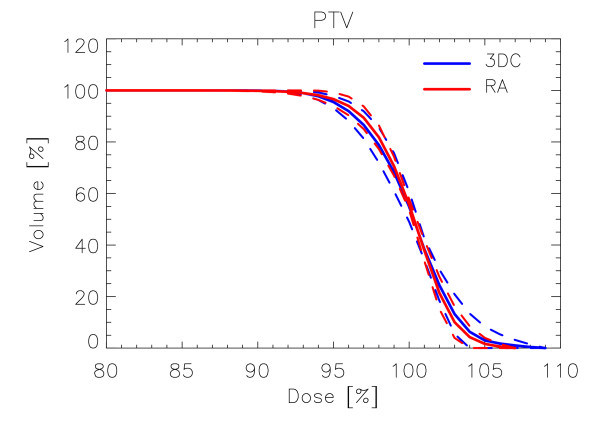
**Average Dose Volume Histograms for PTV for RA and 3DC plans**. Dashed lines represent inter-patient variability at 1 standard deviation.

**Figure 3:**
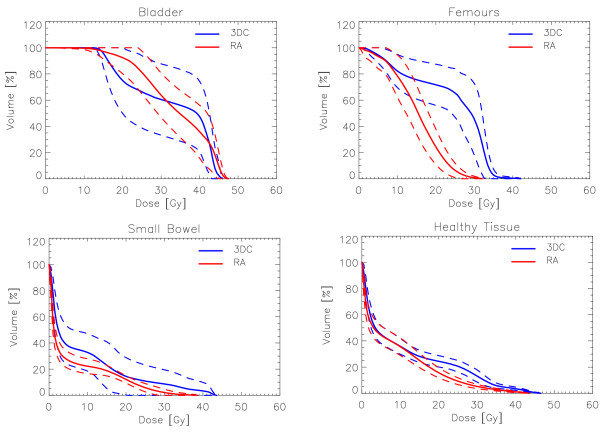
**Average Dose Volume Histograms for Bladder, Bowel, Femurs and Healthy Tissue for RA and 3DC plans**. Dashed lines represent inter-patient variability at 1 standard deviation.

Figure 1 shows examples of dose distributions for two patients treated with RA or with 3DC plans. Colourwash is in the interval from 10 to 47 Gy. PTV, femurs and bladder are outlined as solid lines in the images. Figures 2 and 3 report the average dose volume histograms of the two groups of patients for PTV, bladder, bowels, femurs and healthy tissue. Dashed lines represent the inter-patient variability at one standard deviation.

Table 2 summarises the technical features of the treatment characteristics. Table 3 reports results of the DVH analysis for the primary course of 44/45 Gy. Tables 4 and 5 record the clinical data of the treatments as early acute reactions and surgical outcome whenever available.

**Table 2:** Technical characteristics of RapidArc and conventional plans

	RA	3DC
**Number of arcs or fields**	1 (25/25)	3 (15/20), 4 (5/20)
**Arc length [°]**	358 ± 0.0	NA
**Beam energy**	6 MV (25/25)	6 MV (7/20) 15 MV (13/20)
**Beam on time [min]**	1.24 ± 0.0	0.98 ± 0.2
**Treatment Time [min]**	2.05 ± 0.09	3.42 ± 0.25
**MU**	276 ± 32	293 ± 46
**MU/Gy**	141 ± 14	149 ± 21
**MU/deg**	0.8 ± 0.1	NA
**Dose Rate [MU/min]**	222 ± 25	300
**Gantry speed [deg/sec]**	4.8 ± 0.0	NA
**Collimator angle [°]**	24 ± 8	0 ± 0
**Mean CP area [cm^2^]**	156 ± 28	NA
**Mean field area [cm^2^]**	393 ± 75	249 ± 32
**GAI [%]**	98.33 ± 1.17	NA

MU: monitor units, CP: control point

**Table 3:** Summary of DVH analysis for PTV, Bladder, Femurs, Bowels and healhty tissue.

	RA	3DC	P
	**PTV**		
**Volume [cm^3^]**	1360 ± 250	1358 ± 218	0.98
**Mean [Gy]**	43.9 ± 1.3	44.0 ± 1.0	0.87
**D_1% _[Gy]**	46.1 ± 1.5	46.3 ± 1.4	0.65
**D_5-95% _[Gy]**	3.5 ± 0.8	4.0 ± 0.9	0.10
**D_99% _[Gy]**	41.0 ± 1.3	40.9 ± 0.9	0.75
**V_95% _[%]**	96.5 ± 2.7	95.3 ± 2.5	0.14
**V_107% _[%]**	0.2 ± 0.4	1.0 ± 2.1	0.07
**CI_90%_**	1.2 ± 0.1	1.6 ± 0.1	<0.01
**CI_95%_**	1.1 ± 0.1	1.4 ± 0.1	<0.01
	**Bladder**		
**Volume [cm^3^]**	190 ± 166	98.0 ± 36	0.02
**Mean [Gy]**	34.0 ± 4.2	32.7 ± 7.1	0.45
**V_40 Gy _[cm^3^]**	68.2 ± 38.4	46.5 ± 26.4	0.11
	**Bowels**		
**Volume [cm^3^]**	3417.7 ± 816.5	2609.0 ± 718.1	0.13
**Mean [Gy]**	5.9 ± 1.0	8.7 ± 5.2	0.27
**D_1% _[Gy]**	31.4 ± 4.0	37.8 ± 3.8	0.03
**V_30 Gy _[%]**	2.1 ± 2.6	8.7 ± 10.8	0.22
**V_36 Gy _[%]**	0.4 ± 0.8	4.8 ± 8.3	0.27
	**Femurs**		
**Volume [cm**^3^**]**	322.1 ± 101.2	315.4 ± 74.3	0.81
**Mean [Gy]**	8.7 ± 1.5	10.8 ± 1.7	<0.01
**D_1% _[Gy]**	39.8 ± 1.9	43.9 ± 1.3	<0.01
	**Healthy tissue**		
**Volume [cm^3^]**	24903 ± 6518	21629 ± 3838	0.05
**Mean [Gy]**	8.7 ± 1.5	10.8 ± 1.7	<0.01
**V_10 Gy _[%]**	35.5 ± 6.6	35.8 ± 6.0	0.89
**DoseInt**	2.1 ± 0.5	2.3 ± 0.3	<0.01

D_x% _= dose received by the x% of the volume; V_x% _= volume receiving at least x% of the prescribed dose; CI = ratio between the patient volume receiving at least 95% of the prescribed dose and the volume of the total PTV. DoseInt = Integral dose, [Gy cm^3 ^10^5^]

Data are relative to the primary course of 44/45 Gy only.

**Table 4:** Clinical results after chemo-radiotherapy and surgery.

	TNM	RA	3DC
**Duration of RT [days] (for the 44/45 Gy course)**		32 ± 4 [25-39]	33 ± 7 [28-56]
**Downstaging: (at surgery)**	T	41% (7/17)*	26% (5/19)
	N	12% (2/17)	21% (4/19)
**Upstaging: (at surgery)**	T	6% (1/17)	11% (2/19)
	N	18% (3/17)	11% (2/19)
	M	6% (1/17)	0% (0/19)

* 3 patients are lost to follow-up

Values are reported in % of patients scored for the specific parameter and in brackets the number of patients reporting an effect vs. the total scored).

**Table 5:** Early acute toxicity results.

		RA	3DC
**Erithema**	G1/G2	28%(7/25)	15% (3/20)
	G3/G4	0% (0/25)	0%(0/20)
**Diarrhoea**	G1/G2	40% (10/25)	45% (9/20)
	G3/G4	8% (2/25)	5% (1/20)
**Disuria/incontinency**	G1/G2	0% (0/25)	10%(2/20)
	G3/G4	0%(0/25)	0% (0/20)

From technical features, it is evident how pure beam on time for RA is slightly longer than the corresponding quantity for 3DC but this difference is compensated by the usage of single arc against the usage of multiple static fields with wedges (normally mechanical being oriented in left-right direction and no collimator rotation is applied to keep MLC leaves in the needed direction to generate conformal shapes). This means that a 3DC plan requires a substantially longer time to be delivered. Higher monitor units were observed for 3DC plans because of the usage of wedges. Fixed dose rate of 300 MU/min was selected for 3DC treatments from institutional policy for conventional treatments, while the effective average dose rate for RA plans resulted of ~200 MU/min, associated to a constant maximum gantry speed rotation (dose rate does not affect delivery time for RapidArc as mentioned in the methods). Field size, defined by jaws, resulted larger for RA but, considering the effective aperture size of the continuously adapting control points (elementary apertures), the situation is reversed with RA resulting in smaller average apertures compared to 3DC.

Pre-treatment quality assurances of RA plans resulted in an average gamma agreement index GAI 3% superior to the acceptance threshold of 95% (range: 95.4 - 99.9%), set as references in our institute.

Dosimetric data showed that RA offered a significant improvement in treatment conformality (CI_90% _and CI_95%_) with a trend to significance (p < 0.10) for homogeneity (D_5-95%_) and for V_107%_. No other significant differences between RA and 3DC have been observed. PTV volumes were equivalent between the two groups. Concerning femurs, RA showed a significant reduction in both mean and maximum dose. Bladder data, although a significant difference in the organ's volume was observed (not correlated to any change in patient preparation protocols), no statistically significant differences were observed, with RA delivering low doses to a larger relative volume than 3DC. For bowels, RA presented a systematic additional sparing over the entire dose range but statistically significant only for the maximum significant dose D_1%_.

Significant differences were instead observed for Healthy Tissue with RA showing a lower integral and mean dose.

Concerning clinical outcome, only early results are available. Total treatment duration (including week ends and holidays) resulted in a similar number of days with a wider span for 3DC due to some unscheduled interruptions prolonging the course of treatment for this group. The clinical data showed (Table 4) that, after surgery, a T down-staging was observed in 7 patients over 17 with RA. For 3DC the T down-staging was observed in 5 patients over 19. N down-staging was observed in 2/17 patients in the RA group and 4/19 in the 3DC group of patients. Some up-staging for both T and N were observed in both RA and 3DC patients. Distant metastasis progression was observed in one patient in the RA group and in no patients in the 3DC group.

Chemotherapy is one of the most challenging components of the treatment scheme and was interrupted in a similar proportion of patients between the two groups (12% and 15% respectively) for toxicity. Similar patterns of acute toxicity were observed, as expected, between the two groups. All patients in the 3DC group received chemotherapy while two patients did not received any chemotherapy in the RA group (8%, both post-operative patients).

Concerning quantitative results on acute toxicity (table 5), about 50% of patients showed diarrhoea up to grade 3 (G3 in the 8% of RA patients and 5% in the 3DC patients). 28% of RA patients manifested local erithema of grade 1 or 2, 15% in the 3DC group of patients; no grade 3 erithema or higher were observed. Two patients in the 3DC group developed disuria/incontinency, none for RA.

## Discussion and conclusions

Based on the results of an intensive program of pre-clinical investigations performed at planning level [12-18] to assess its reliability and potential benefit, RA, a Volumetric Modulated Arc Therapy, was introduced in clinical practice since September 2008 at our institute for a variety of indications. The present study reports about the early findings from the treatment of a group of 25 patients affected by advanced rectal adenocarcinoma irradiated with RA. Most of the patients received concomitant chemotherapy with capecitabine in a pre-operative neo-adjuvant scheme.

The main objective of the first phase of clinical introduction of RA is the assessment of the possibility to administer to patients treatments not inferior and possibly superior to previously adopted conventional modalities. These results should be achieved without introducing elements of potential confusion like alterations of the fractionation schemes (acceleration or hypo-fractionation for example) or like the attempt to maximise dosimetric performances (e.g. enhanced protection of organs at risk). Further studies will assess the elements of improvement once the safety of the new approach is consolidated in routine practice.

Under this perspective, RA showed, according to the here presented data, the capability to reliably reproduce the dosimetric quality of conventional conformal plans, previously used as the standard of treatment for this class of patients, with some already observable improvement in i) conformality of treatments, ii) reduction of hot spots inside target volume, iii) reduction of distant organs at risk involvement like femurs or bowels, iv) global reduction of healthy tissue involvement. Although most of the trends observed and reported in the tables are minor, not statistically significant and likely not clinically relevant, the three points mentioned above (statistically significant) suggest how the advanced technique with volumetric modulation with arcs might be beneficial and confirm general findings from in-silico investigations [12-18]. The dosimetric improvement achieved with RA compared to 3DC is quite obvious and might be similar to what achievable with other IMRT approaches. In our institute the selection of RA instead of, e.g., fixed gantry IMRT was based also on logistic issues as discussed below.

These results enabled the activation of a second phase, aiming to push RA towards improved sparing of organs at risk, particularly the bladder and the bowels, as already potentially proven in planning investigation for cervix uteri treatments [13].

Having achieved the same quality of treatments of previously adopted techniques, RA confirmed also some advantages at a logistical level. It allowed a significant reduction of effective treatment time, defined as the time needed to deliver a single fraction with the exclusion of time needed to position the patients and to acquire data for image guidance. The measured treatment time reduction with RA was about 40%. If the absolute benefit is a reduction of about 1.4 minutes which seems to be limited, it shall be noted that this was obtained from a comparison against a 3DC technique rather than IMRT with fixed gantry fields. IMRT might allow abetter dosimetric quality but at the expense of prolonged treatment time. In addition, RA allows avoidance of usage of wedges. Mechanical wedges were used in our institute instead of dynamic wedges to allow useful orientation of them without conflicting with MLC leaves movement, but this required manual operation by radiographers with multiple entries into in the treatment room. Therefore, the logistic benefit derived from RA is significant for a department and reduces also the risk of human error.

From a more general perspective, optimization and calculation of RA plans is obviously a longer process than the forward calculation of 3DC plans in most of the cases. Nevertheless, although the number of treatment machines in a department cannot be easily increased, the planning power of a radiotherapy facility is much more easily customizable and addition of more planning station, availability of faster hardware and distribution of calculation burden over the planning network are all methods of ''adaptation" achievable at reasonable costs. Therefore, the longer time objectively needed for any IMRT planning process, particularly for RA, has a potentially smaller impact on the clinical throughput than the time-slots to be allocated per patients at the treatment units.

From the clinical point of view, data presented here show that RA can be considered as a safe modality for this category of patients with a good potential for improving OAR sparing and acute toxicity. On the clinical outcome, although interesting, it is possible that the observed increased in down-staging is due to statistical fluctuations from the very small group of patients. In theory, having not introduced any fractionation alteration (like dose escalation) an improved downstaging is not expected and should be investigated on larger samples. To notice anyway that the downstaging rate observed for patients treated with RA is consistent with reported data [3]. The possibility of improving organs at risk sparing shall be explored with RA in order to compensate or mitigate known negative effects from concomitant chemotherapy (e.g. reduction of diarrhoea). The smoother process of RA and its potential reduction in acute toxicity could also lead to a more uniform duration of treatments reducing the risk of unscheduled and undue interruptions.

It is obvious that the present study cannot be considered as conclusive and that long term observation of patients is needed to measure outcome and late toxicity. These preliminary results are anyway encouraging for further experience in this field.

In summary, twenty-five patients with advanced rectal cancer were treated preoperatively within and neoadjuvant radio-chemotherapy scheme with radiation course delivered with Volumetric Modulated Arc Therapy according to the RapidArc^® ^implementation in a clinical feasibility protocol. Quality of treatments resulted comparable with conformal modality used for benchmarking with improved conformality and reduced treatment times. Clinical outcome and early acute toxicity and assessment of tumour stage at surgery showed similar results. Anyway this study was not comparative and the study is under power to draw significant conclusion. Future investigations will aim to increase sparing of organs at risk and to look to long term outcome having the first phase achieved the primary goal to demonstrate safety and efficacy of RA.

## Competing interests

LC acts as Scientific Advisor to Varian Medical Systems and is Head of Research and Technological Development to Oncology Institute of Southern Switzerland, IOSI, Bellinzona.

No special competing interest exists for any other author.

## Authors' contributions

AF: study coordination, manuscript preparation

LC: study coordination, manuscript preparation

GN: supervision of planning, manuscript proof

EV: physics data collection and analysis

AC: physics data collection and analysis

AR: patient accrual, management and data collection, manuscript proof and study coordination

GP: patient accrual, management and data collection, manuscript proof

MS: patient accrual, management and data collection, manuscript proof

All authors approved the final manuscript
